# What is the optimum time for initiation of early mobilization in mechanically ventilated patients? A network meta-analysis

**DOI:** 10.1371/journal.pone.0223151

**Published:** 2019-10-07

**Authors:** Nannan Ding, Zhigang Zhang, Caiyun Zhang, Li Yao, Liping Yang, Biantong Jiang, Yuchen Wu, Lingjie Jiang, Jinhui Tian

**Affiliations:** 1 School of Nursing, Lanzhou University, Lanzhou, China; 2 Intensive Care Unit, The First Hospital of Lanzhou University, Lanzhou, China; 3 Department of Nursing, The First Hospital of Lanzhou University, Lanzhou, China; 4 Evidence-Based Medicine Center, Lanzhou University, Lanzhou, China; All India Institute of Medical Sciences, Bhopal, INDIA

## Abstract

Early mobilization has been proven to be an effective and safe intervention for preventing complications in mechanically ventilated patients; however, there is currently no unified definition of the optimal mobilization initiation time, hindering widespread clinical implementation. As clinicians are increasingly aware of the benefits of early mobilization, the definition of early mobilization is important. The purpose of this study was to evaluate the effects of different early mobilization initiation times on mechanically ventilated patients and rank these times for practical consideration. The Chinese Biomedical Literature Database, the Chinese Knowledge Infrastructure, Wanfang Data, PubMed, Cochrane Library, Web of Science, and Embase databases, along with grey literature and reference lists, were searched for randomized control trials (RCTs) that evaluated the effects of early mobilization for improving patient outcomes; databases were searched from inception to October 2018. Two authors extracted data independently, using a predesigned Excel form, and assessed the quality of included RCTs according to the Cochrane Handbook (v5.1.0). Data were analyzed using Stata (v13.0) and Review Manager (v5.3.0). A total of 15 RCTs involving 1726 patients and seven mobilization initiation times (which were all compared to usual care) were included in our analysis. Network meta-analysis showed that mechanical ventilation for 48–72 h may be optimal to improve intensive care unit acquired weakness (ICU-AW) and reduce the duration of mechanical ventilation; however, there were no significant differences in length of ICU stay according to mobilization initiation time. The results of this study indicate that initiation of mobilization within 48–72 h of mechanical ventilation may be optimal for improving clinical outcomes for mechanically ventilated patients.

## Introduction

Advances in medical technology and instrumentation in intensive care units (ICUs) have resulted in clear increases in patient survival rates; however, long-term immobility and bed rest can lead to physical dysfunction, such as ICU-acquired weakness (ICU-AW) and prolonged mechanical ventilation [1–2]. Further, complications of these conditions can persist for more than 5 years after hospital discharge, hindering the return of patients to normal social function [3].

Early mobilization can improve patient muscle strength, reduce the duration of mechanical ventilation, and improve patient quality of life, and is associated with low adverse event rates (<1%) [4–7]; however, to date, the time of early mobilization initiation has not been standardized. Harrold [8] conducted a systematic review of the time of early mobilization initiation in patients classified according to three criteria, which was ICU patients with or without mechanical ventilation or non-ICU patients. Clarissa et al. [9] conducted a systematic review of definitions of early mobilization for mechanically ventilated patients, which included 76 studies, and suggested that the time of initiation should be defined by mechanical ventilation duration, and demonstrated that an agreed standardized definition is a prerequisite to advance research into, and practice of early mobilization.

Initiation times range from ≤ 24 h after mechanical ventilation to ≥ 1 week after ICU admission. Morris et al. [10] produced the first report of early mobilization of ICU patients, with initiation within 48 h of mechanical ventilation. Further, one study [11] showed that mobilization started at ≥ 7 days after ICU admission cannot improve physical function or medical outcomes. The UK National Institute for Health and Clinical Excellence (NICE) recommends that mobilization should start as early as possible during an ICU stay [12]. At present, ICU medical staff are increasingly aware of the benefit of early mobilization for mechanically ventilated patients; however, when mobilization should be initiated remains unclear and controversial, which hinders the widespread implementation of this practice in the clinic. Therefore, exploration of the appropriate time to initiate mobilization is of great significance.

Traditional meta-analyses have demonstrated that early mobilization can improve physical function; however, there have been no studies regarding the effect of different initiation times or direct comparisons of different initiation times. Network meta-analysis (NMA) allows indirect comparisons of different interventions (without requiring direct comparisons), and selection of the optimal solution [13,14]. Therefore, this study aimed to evaluate the effects of different early mobilization initiation times in mechanically ventilated patients and to rank the different initiation times for practical application using an NMA approach. Our findings support the initiation of mobilization within 48–72 h of mechanical ventilation.

## Materials and methods

This meta-analysis was conducted based on the Preferred Reporting Items for Systematic Reviews and Meta-Analyses (PRISMA) NMA Checklist [15] and the Cochrane Handbook for Systematic Reviews (v5.1.0) [16]. No ethics approval was required.

### Inclusion/Exclusion criteria

The included population were aged > 18 years and had undergone mechanical ventilation. The intervention groups underwent early mobilization, initiated at various time points, as follows: within ≤ 24h, 24–48h, 48–72h, 72–96h, and > 96 h of mechanical ventilation, and > 5 and > 7 days after ICU admission. Control groups received usual nursing care. Outcomes included ICU-AW (according to Medical Research Council (MRC) diagnosis criteria [17]), duration of mechanical ventilation, and length of ICU stay. Studies were randomized control trials (RCTs) published in English and Chinese.

Exclusion criteria were as follows: (1) abstracts, letters, case reports, non-RCTs, expert opinions and reviews, and repeated literature; and (2) studies that did not specify the time of mobilization initiation or related outcomes.

### Data sources and search strategies

The Chinese Biomedical Literature Database (CBM), Chinese Knowledge Infrastructure (CNKI), Chinese Wanfang Data, PubMed, Cochrane Library, Web of Science, and Embase databases were searched from inception to October 2018, along with grey literature and reference list searches. The search strategies were as follows: “early activit* OR accelerated ambulation* OR early action* OR early motion* OR early mobilisation* OR active* in early stage OR early-stage activit* OR early ambulant* OR early movement*” AND “artificial respiration OR mechanical ventilation” AND “randomized controlled trial* OR RCT*”. The search strategy are provided in Appendix 1–7.

### Study selection

All studies retrieved were imported into literature management software (EndNote X8). To screen studies, two authors independently reviewed the titles and abstracts, and then reviewed the full-text of the included studies for quantitative analysis. A third reviewer resolved any discrepancies between the two authors. Also, two authors independently extracted the data, according to a predesigned form, which included study characteristics (first author, year, country, ICU type), patient characteristics (sample size, male/female, age, interventions, and controls), and outcomes (ICU-AW, duration of mechanical ventilation, and length of ICU stay).

### Quality assessment

Two reviewers independently assessed the risk of bias using the Cochrane Handbook for Systematic Reviews (v5.1.0), which includes seven domains (random sequence generation, allocation concealment, participants/personnel blinding, outcome assessor blinding, incomplete outcome data, selective reporting, and other bias [16]).

## Statistical analysis

NMA was performed using the mvmeta package in Stata (v13.0). Comparisons between different initiation times were represented using network plots, with the lines between dots representing direct comparisons and line thickness representing the quantity of comparisons between two interventions, i.e., the sample size involved in each comparison. Both comparisons of dichotomous variables and continuous variables are presented as mean with 95% confidence interval (95% CI); but dichotomous variables show the index number and continuous variables are logarithm. Ranking probabilities were estimated by determining the surface under the cumulative ranking (SUCRA) value, where higher SUCRA values indicate a higher probability of superior ranking. Funnel plots were constructed to represent publication bias, where symmetrical distribution indicates an absence of publication bias. The risks of bias of the included studies were analyzed using Review Manager (v5.3.0), where green, yellow, and red in the image represent low, unclear, and high risk of bias, respectively.

### Literature search

**Fig 1:**
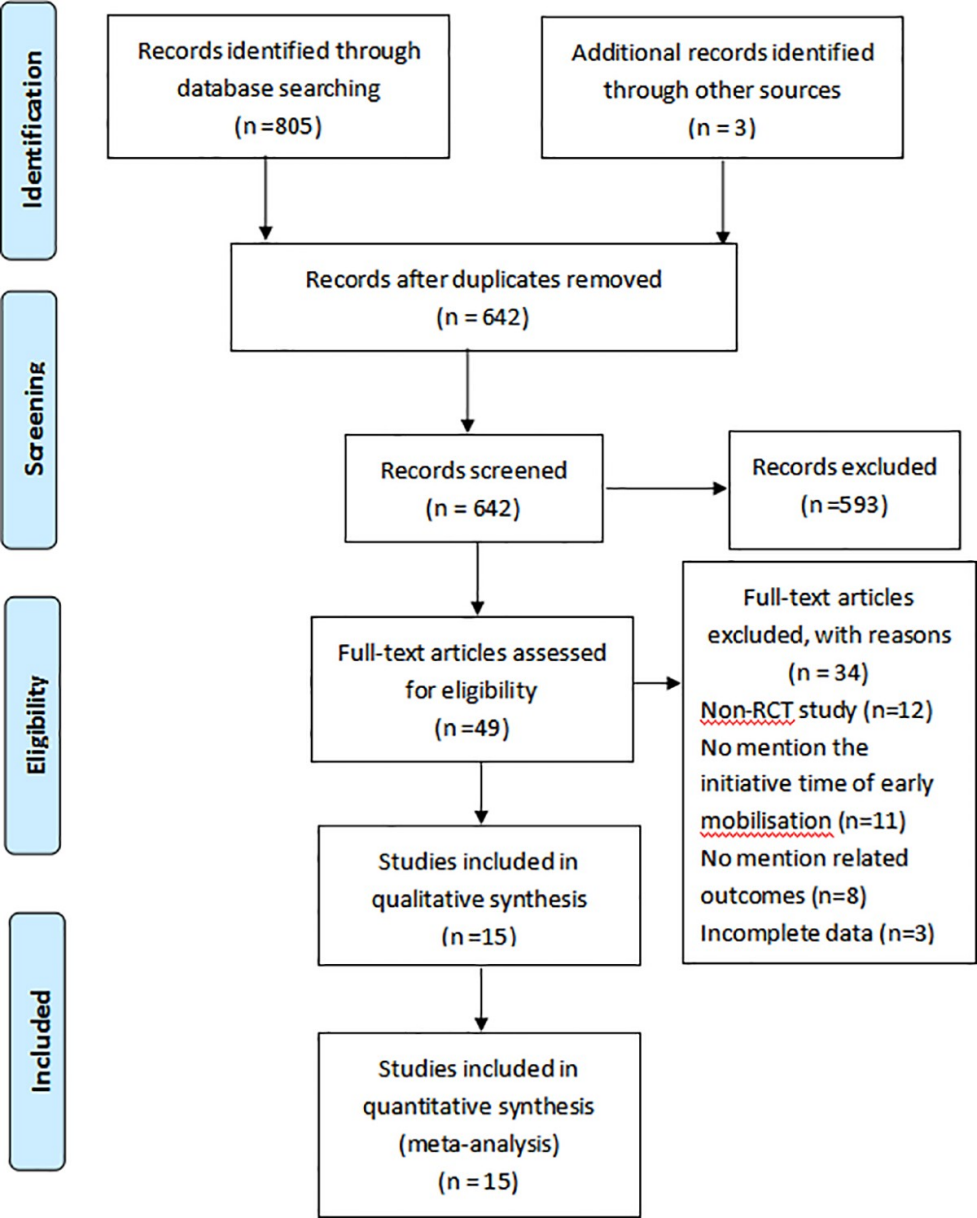
RCT: Randomized control trial.

A total of 808 studies were initially retrieved from electronic databases, including studies 625 in English and studies 183 in Chinese. There were 642 studies remaining after exclusion of duplicates using EndNote X8 and manual exclusion. There were 34 studies excluded by some reasons, for example, non-RCTs (n = 12), no mention the initiative time of mobilization (n = 11), no mention related outcomes (n = 8), incomplete data (n = 3). Finally, 15 studies were included in the analysis. The study flow diagram is shown in Fig 1.

### Study and patient characteristics

A total of 15 studies were included, including seven published in Chinese and eight in English, with 1726 patients (868 and 858 in the intervention and control groups, respectively). There were seven initiation time categories (within ≤ 24h, 24–48h, 48–72h, 72–96h, and > 96 h of mechanical ventilation; and > 5 and > 7 days after ICU admission) along with usual care. All 15 studies provided data that allowed indirect comparisons of the initiation times, with no direct comparisons in any individual study. Basic study characteristics are presented in S1 Table

## Results

### Quality assessment

**Fig 2:**
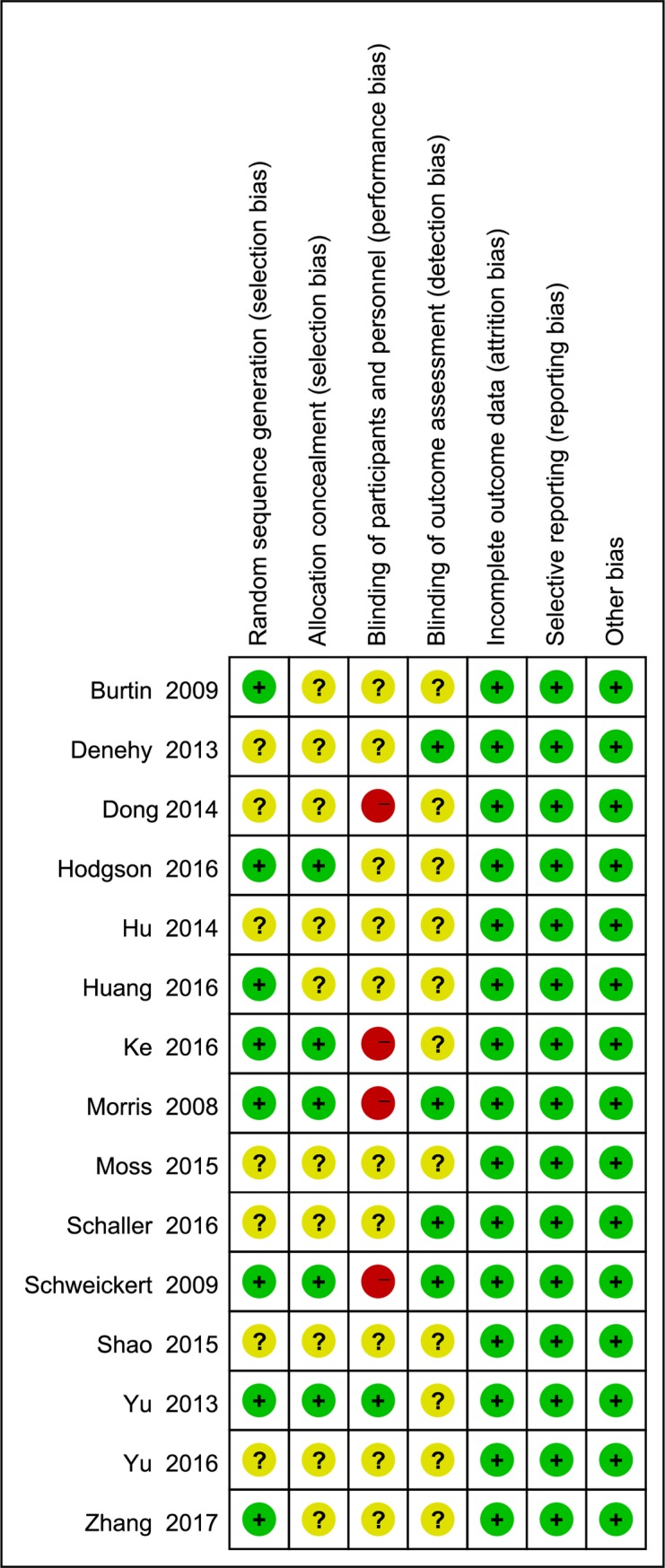
Green: Low risk of bias; Yellow: Unclear risk of bias; Red: High risk of bias.

In Fig 2, green, yellow, and red represent low, unclear, and high risk of bias respectively. Eight studies [4,10,18,21,23,27–29] were adequately randomized, five [4,10,18,27,29] reported allocation concealment, and four [4,10,19,22] reported blinding of outcome assessment. Four studies [4,10,20,27] did not report blinding of participants and personnel. All other risks of bias were low.

### Network plots of included studies

**Fig 3:**
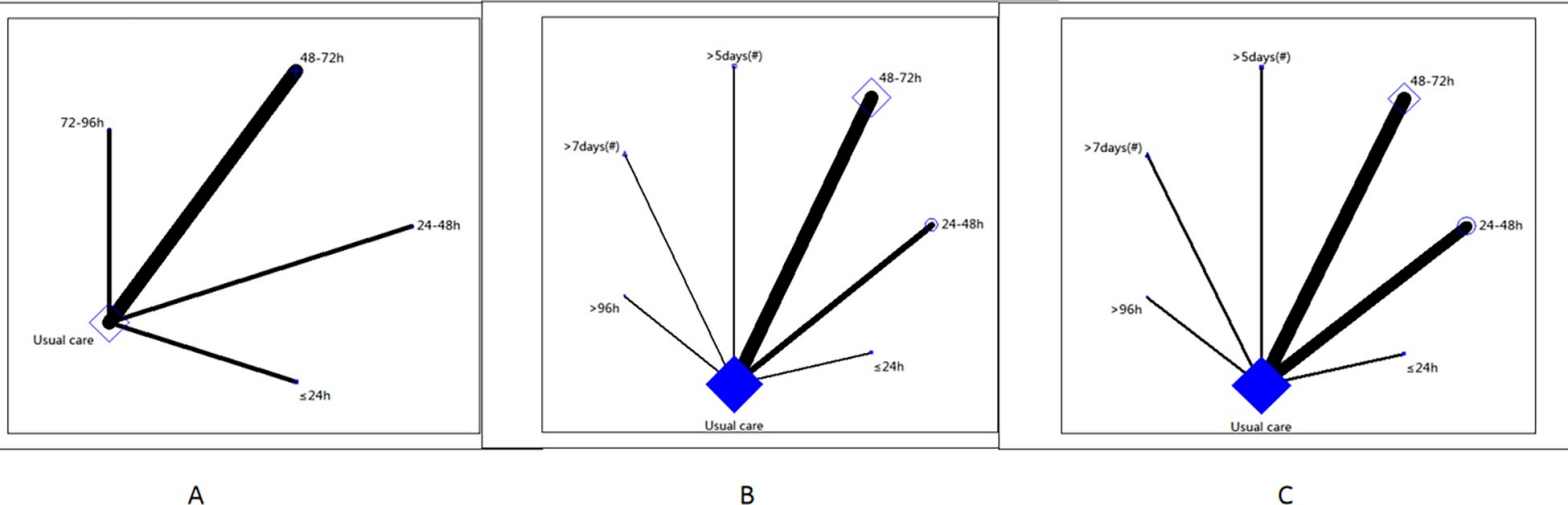
Network plot of included studies. A: Network plot of ICU acquired weakness. B: Network plot of duration of mechanical ventilation. C: Network plot of ICU length of stay.

The control in all of the studies was usual care. ICU-AW data were included in six studies [4,19,23,27–29]. Three of these studies [4,27,29] investigated mobilization within 48–72 h of mechanical ventilation and one study each investigated mobilization within ≤ 24h [28], 24–48h [19], and 72–96 h [23] of mechanical ventilation (Fig 3A). Data on the duration of mechanical ventilation were included for 13 studies [4,10,11,18,20–22,24–29]. Six of these studies [4,20,24,25,27,29] investigated mobilization within 48–72 h of mechanical ventilation, three studies [10,18,26] investigated mobilization within 24–48 h of mechanical ventilation, and one study each investigated mobilization within ≤ 24h [28] or > 96 h [11] of mechanical ventilation or > 5 [22] or > 7 days [21] after ICU admission (Fig 3B). Data on ICU length of stay were available for 13 studies [4,10–11,18–22,24,26–29]. Four of these studies [4,20,24,29] investigated mobilization within 48–72 h of mechanical ventilation, four studies [10,18–19,26] investigated mobilization within 24–48 h of mechanical ventilation, and one study each investigated mobilization within ≤ 24h [28] or > 96 h [11] of mechanical ventilation or > 5 [22] or > 7 days [21] after ICU admission (Fig 3C).

### Forest plots

**Fig 4:**
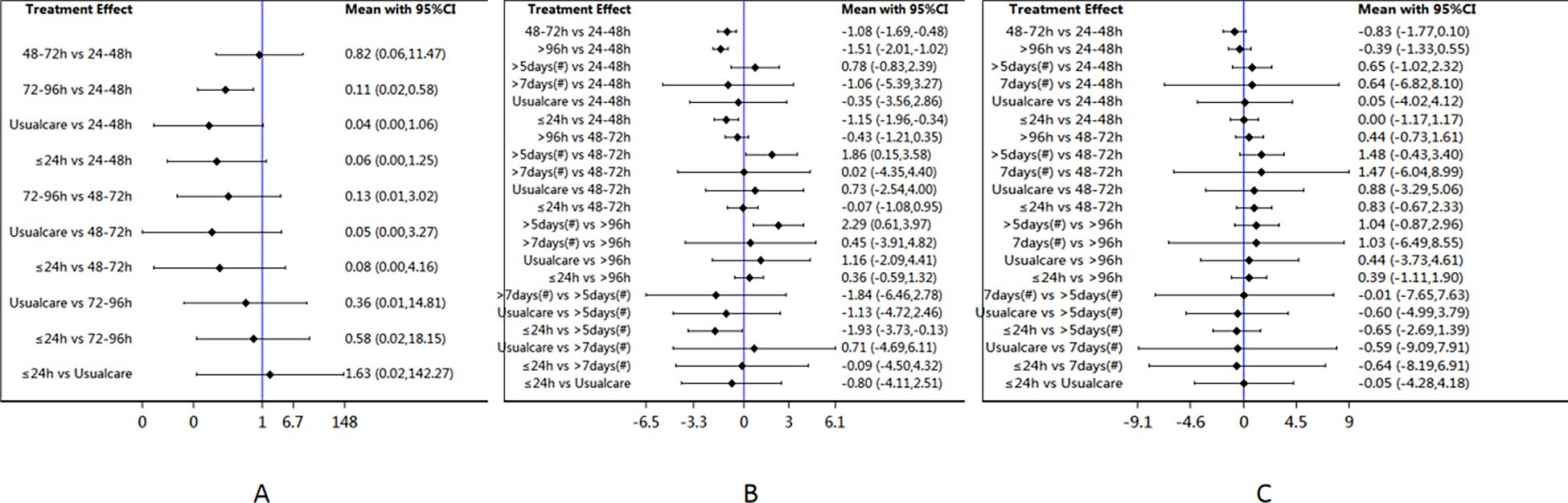
95%CI: 95% Confidence Interval; (#): ICU admitted time.

NMA of data on the incidence of ICU-AW showed that there was a significant difference between mobilization within 72–96 h and 24–48 h of mechanical ventilation, with the former leading to a greater reduction in ICU-AW (Fig 4A). No significant differences were detected for the other comparisons. NMA of data on the duration of mechanical ventilation revealed significant differences between mobilization within ≤ 24 h, 48–72 h, > 96 h, and 24–48 h of mechanical ventilation, with shorter durations for patients mobilized at ≤ 24h, 48–72h, and > 96 h relative to 24–48 h. Further, there were significant differences between mobilization within ≤ 24 h or > 96 h of mechanical ventilation and > 5 days after ICU admission, with ≤ 24 h or > 96 h leading to shorter durations (Fig 4B). There were no significant differences in length of ICU stay among the seven initiation times (Fig 4C).

### Ranking

**Fig 5:**
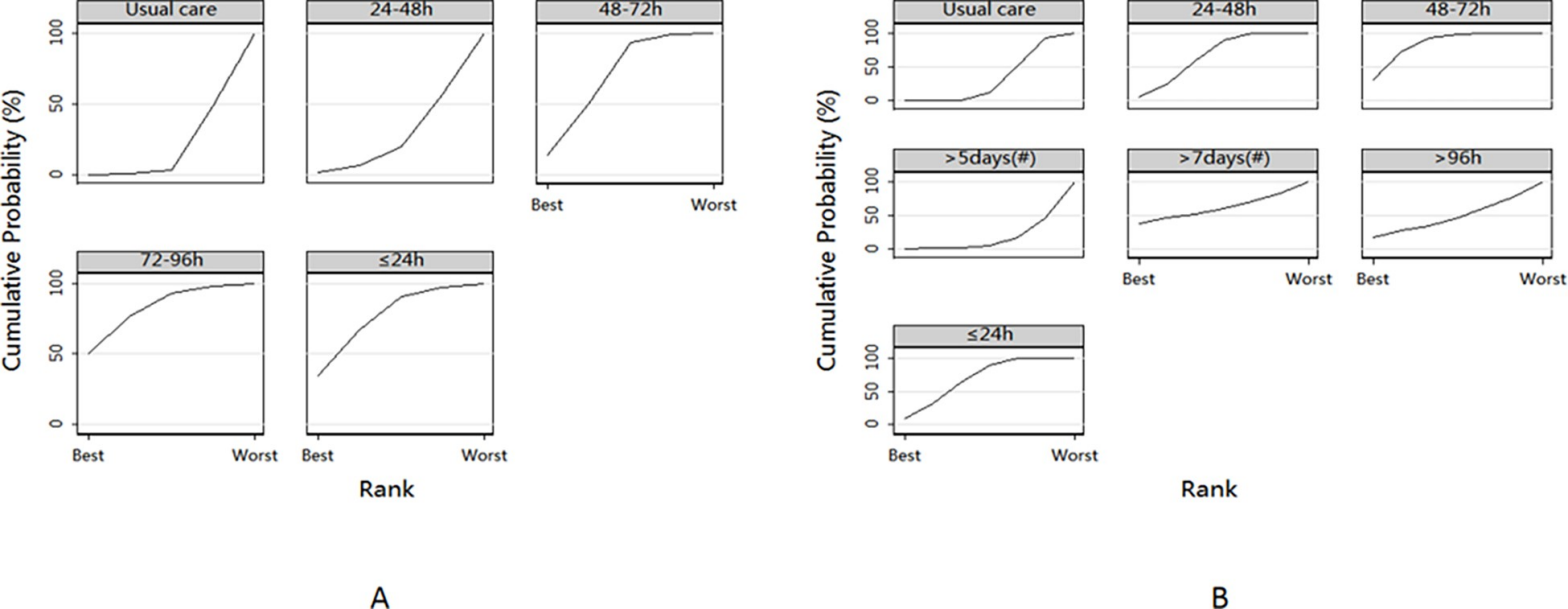
SUCRA: Surface under the cumulative ranking; (#): ICU admitted time.

Larger SUCRA values indicate that a specific early mobilization time is superior to another. The best initiation time for reducing ICU-AW after mechanical ventilation, was 72–96 h, followed by 48–72 h. To decrease the duration of mechanical ventilation, mobilization at 48–72 h, followed by ≤ 24 h, may be the best initiation times (S2 Table). Overall, based on the forest plots and SUCRA data, 48–72 h after mechanical ventilation may be the best time to initiate mobilization (Fig 5A and 5B; S2 Table).

### Publication bias

**Fig 6:**
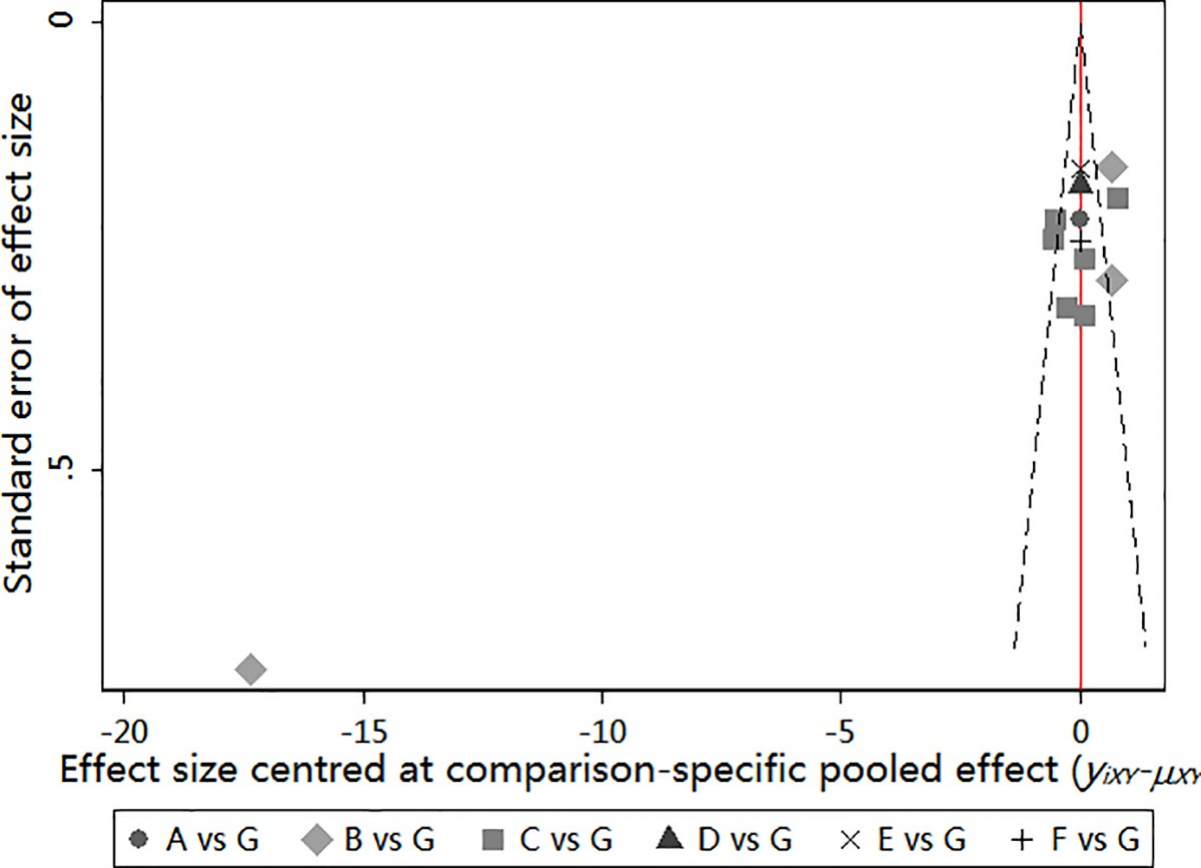
A: mechanical ventilation ≤ 24h; B: mechanical ventilation with 24h-48h; C: mechanical ventilation with 48h-72h; D: mechanical ventilation > 96h; E: ICU admitted > 5days; F: ICU admitted > 7days; G: Usual care.

A funnel plot was constructed to analyze the publication bias among the included studies. If the symmetrical distribution of studies around the red line indicates no publication bias or a small sample effect (Fig 6). However, there is one data point far away from other studies, which indicate there is publication bias or small sample effect of this study.

### Inconsistency between direct and indirect comparisons

There were no available loops formed by the study arms; therefore, loop-specific tests were not performed.

## Discussion

The results of our NMA demonstrate that early mobilization, started within 72–96 h of mechanical ventilation, may be optimal for decreasing ICU-AW, while initiation within 48–72 h of mechanical ventilation may be superior for decreasing mechanical ventilation duration. Overall, based on our confidence interval and ranking results, we conclude that early mobilization initiated within 48–72 h of mechanical ventilation is likely optimal for improving the outcomes of mechanically ventilated patients. Of the 15 RCTs included in this study, five reported allocation concealment, four reported blinding of outcome assessment, and four studies did not report blinding of participants and personnel which showed that there were high risk bias of these four studies. The funnel plot of the studies was symmetrical, other than one study. Which may be of Hodgson et al [18], because of the small sample included; may be from Schaller et al [19], due to the patients included were surgical patients that wean from mechanical ventilation earlier; and it may because of Shao [26] which the study was from different country.

ICU-AW is defined as clinically detectable weakness, without possible cause other than critical illness in ICU patients [30]. ICU-AW is an independent predictor of prolonged mechanical ventilation [31–32] and prevention of ICU-AW can reduce the duration of mechanical ventilation. Muscle strength drops by 3% to 11% for each additional day of immobility [33], and when it reaches a 40% reduction, the rate of patient mortality significantly increases. Further, rates of ICU-AW can be as high as 33–82% when mechanical ventilation continues for 4–7 days [34–36].

The results of this NMA show that it may be optimal to mobilize patients within 48–72 h of mechanical ventilation. Another study [18] showed that earlier mobilization resulted in superior rehabilitation; however, the clinical implementation of early mobilization has been limited because of various factors, including respiratory and hemodynamic instability and patient safety concerns [37–39]. Generally, the period within several hours of mechanical ventilation is the acute disease phase, where respiratory and hemodynamic parameters are unstable, meaning that the safety of patients cannot be guaranteed. Therefore, most clinicians choose to mobilize patients at a later time, which can prevent ICU-AW and reduce adverse events. Overall, mobilization initiation within 48–72 h of mechanical ventilation may be optimal.

## Limitations of this study

This study had several limitations. First, despite a comprehensive search of Chinese and English databases, few RCTs met the inclusion criteria; therefore, the number of included studies was small, and the quality of the studies was not high, which may have influenced the results of our analyses. Second, some studies did not report blinding and allocation concealment, which may have led to exaggeration of clinical effects; meanwhile, the funnel plot analysis indicated publication bias or a small sample effect. Third, the designs of the included early mobilization studies were similar; however, the frequency, type, and intensity of early mobilization differed among them, which may have influenced the results of this study. Finally, the usual nursing practices applied for the control groups in the included studies may have differed, which could have introduced heterogeneity and influenced the results of this study.

## Conclusion

We conclude that mobilization within 48–72 h of mechanical ventilation may be optimal for improvement of clinical outcomes. Based on our data, we suggest the following: 1) In clinical practice, clinical staff should choose an initiation time appropriate for their specific ICU; 2) In the future, researchers should conduct direct comparisons between different initiation times; for example, comparisons between mobilization within or after 48 h of mechanical ventilation, or at other times; 3) Research should be conducted to assess the long-term outcomes of different early mobilization initiation times; for example, investigation of mortality rates 1, 3, and 12 months after discharge.

## Supporting information

S1 Appendix PubMed search strategy. (DOCX)

S2 Appendix Cochrane library search strategy. (DOCX)

S3 Appendix Web of science search strategy. (DOCX)

S4 Appendix Embase search strategy. (DOCX)

S5 Appendix CBM search strategy. (DOCX)

S6 Appendix CNKI search strategy. (DOCX)

S7 Appendix Wanfang data search strategy. (DOCX)

S1 Table Basic characteristics of included studies. USA, United States of America; COPD, chronic obstructive pulmonary disease; ICU, intensive care unit; SICU, surgical intensive care unit; MICU, medical intensive care unit; A, < 24 h after mechanical ventilation; B, 24–48 h after mechanical ventilation; C, 48–72 h after mechanical ventilation; D, 72–96 h after mechanical ventilation; E, > 96 h after mechanical ventilation; F, > 5 days after ICU admission; G, > 7 days after ICU admission; H, usual care; MRC, Medical Research Council; 1, ICU acquired weakness; 2, duration of mechanical ventilation; 3, length of ICU stay.
(DOCX)

S2 Table SUCRA and Meanrank results. SUCRA, surface under the cumulative ranking; ICU,Intensive care unit;—, not mentioned.
(DOCX)

S1 File PRISMA checklist. (DOCX)
