# Supplementary material for: What is the optimum time for initiation of early mobilization in mechanically ventilated patients? A network meta-analysis
Source: PLoS One. 2019 Oct 7;14(10):e0223151. doi: 10.1371/journal.pone.0223151 (PMC6779259; doi:10.1371/journal.pone.0223151)
Supplement: S1 Appendix — (DOCX) [file pone.0223151.s001.docx]

Appendix 1 PubMed search strategy

#1 "early ambulation"[Mesh]

#2 early activity[Title/Abstract] OR accelerated ambulation[Title/Abstract] OR early action[Title/Abstract] OR early motion[Title/Abstract] OR early mobilisation[Title/Abstract] OR active in early stage[Title/Abstract] OR early-stage activity[Title/Abstract] OR early ambulant[Title/Abstract] OR early movement[Title/Abstract]

#3 #1 OR #2

#4 "respiration, artificial"[Mesh]

#5 artificial respiration[Title/Abstract] OR mechanical ventilation[Title/Abstract]

#6 #4 OR #5

#7 "randomized controlled trial"[Mesh]

#8 randomized controlled trial[Title/Abstract] OR RCT[Title/Abstract]

#9 #7 OR #8

#10 #3 AND #6 AND #9
