# Supplementary material for: What is the optimum time for initiation of early mobilization in mechanically ventilated patients? A network meta-analysis
Source: PLoS One. 2019 Oct 7;14(10):e0223151. doi: 10.1371/journal.pone.0223151 (PMC6779259; doi:10.1371/journal.pone.0223151)
Supplement: S2 Appendix — (DOCX) [file pone.0223151.s002.docx]

Appendix 2 Cochrane Library search strategy

#1 (early activity):ti,ab,kw OR (accelerated ambulation):ti,ab,kw OR (early action):ti,ab,kw OR (early motion):ti,ab,kw OR (early mobilisation):ti,ab,kw OR (active in early stage):ti,ab,kw OR (early-stage activity):ti,ab,kw OR (early ambulant):ti,ab,kw OR (early movement):ti,ab,kw

#2 (artificial respiration):ti,ab,kw OR (mechanical ventilation):ti,ab,kw

#3 (randomized controlled trial):ti,ab,kw

#4 #1 AND #2 AND #3
