# Supplementary material for: What is the optimum time for initiation of early mobilization in mechanically ventilated patients? A network meta-analysis
Source: PLoS One. 2019 Oct 7;14(10):e0223151. doi: 10.1371/journal.pone.0223151 (PMC6779259; doi:10.1371/journal.pone.0223151)
Supplement: S3 Appendix — (DOCX) [file pone.0223151.s003.docx]

Appendix 3 Web of Science search strategy

#1 TS=(early activity OR accelerated ambulation OR early action OR early motion OR early mobilisation OR active in early stage OR early-stage activity OR early ambulant OR early movement)

#2 TS=(artificial respiration OR mechanical ventilation)

#3 TS=randomized controlled trial

#4 #1 AND #2 AND #3
