# Supplementary material for: What is the optimum time for initiation of early mobilization in mechanically ventilated patients? A network meta-analysis
Source: PLoS One. 2019 Oct 7;14(10):e0223151. doi: 10.1371/journal.pone.0223151 (PMC6779259; doi:10.1371/journal.pone.0223151)
Supplement: S4 Appendix — (DOCX) [file pone.0223151.s004.docx]

Appendix 4 Embase search strategy

#1 early activity OR accelerated ambulation OR early action OR early motion OR early mobilisation OR active in early stage OR early-stage activity OR early ambulant OR early movement

#2 artificial respiration OR mechanical ventilation

#3 randomized controlled trial OR RCT

#4 #1 AND #2 AND #3
