# Supplementary material for: What is the optimum time for initiation of early mobilization in mechanically ventilated patients? A network meta-analysis
Source: PLoS One. 2019 Oct 7;14(10):e0223151. doi: 10.1371/journal.pone.0223151 (PMC6779259; doi:10.1371/journal.pone.0223151)
Supplement: S5 Appendix — (DOCX) [file pone.0223151.s005.docx]

Appendix 5 Chinese biomedical literature database search strategy

#1 早期运动[常用字段] OR 早期下床活动[常用字段] OR 早期功能锻炼[常用字段] OR 早期运动训练[常用字段] OR 早期主动活动[常用字段] OR 早期物理治疗[常用字段]

#2 机械通气[常用字段] OR 肺通气[常用字段] OR 通气机[常用字段]

#3 对照试验[常用字段] OR 随机对照试验[常用字段]

#4 #1 AND #2 AND #3

#1 early mobility[Common field] OR early mobilization out of bed[Common field] OR early function rehabilitation[Common field] OR early exercise training[Common field] OR early active mobilization[Common field] OR early physical therapy[Common field]

#2 mechanical ventilation[Common field] OR Lung ventilation[Common field] OR ventilator

#3 randomized controlled trial[Common field] OR RCT[Common field]

#4 #1 AND #2 AND #3
