# Supplementary material for: What is the optimum time for initiation of early mobilization in mechanically ventilated patients? A network meta-analysis
Source: PLoS One. 2019 Oct 7;14(10):e0223151. doi: 10.1371/journal.pone.0223151 (PMC6779259; doi:10.1371/journal.pone.0223151)
Supplement: S6 Appendix — (DOCX) [file pone.0223151.s006.docx]

Appendix 6 Chinese knowledge infrastructure search strategy

#1 早期运动OR 早期下床活动 OR 早期功能锻炼 OR 早期运动训练 OR 早期主动活动 OR 早期物理治疗

#2 机械通气 OR 肺通气 OR 通气机

#3 对照试验 OR 随机对照试验

#4 #1 AND #2 AND #3

#1 early mobility OR early mobilization out of bed OR early function rehabilitation OR early exercise training OR early active mobilization OR early physical therapy

#2 mechanical ventilation OR Lung ventilation OR ventilator

#3 randomized controlled trial OR RCT

#4 #1 AND #2 AND #3
