# Supplementary material for: What is the optimum time for initiation of early mobilization in mechanically ventilated patients? A network meta-analysis
Source: PLoS One. 2019 Oct 7;14(10):e0223151. doi: 10.1371/journal.pone.0223151 (PMC6779259; doi:10.1371/journal.pone.0223151)
Supplement: S7 Appendix — (DOCX) [file pone.0223151.s007.docx]

Appendix 7 Wanfang data search strategy

(主题:("早期运动") + 主题:("早期下床活动") + 主题:("早期功能锻炼") + 主题:("早期运动训练") + 主题:("早期主动活动") + 主题:("早期物理治疗")) * (主题:("机械通气") + 主题:("肺通气") + 主题:("通气机"))

(Topic:(“early mobilization”) + (Topic:(“early mobilization out of bed”) +(Topic:(“early function rehabilitation”) +(Topic:(“early exercise training”) +(Topic:(“early active mobilization”) +(Topic:(“early physical therapy”)) * ((Topic:(“mechanical ventilation”) +(Topic:(“Lung ventilation”) +(Topic:(“ventilator”) ) * ((“randomized controlled trial”) + (“RCT”))
