# Supplementary material for: What is the optimum time for initiation of early mobilization in mechanically ventilated patients? A network meta-analysis
Source: PLoS One. 2019 Oct 7;14(10):e0223151. doi: 10.1371/journal.pone.0223151 (PMC6779259; doi:10.1371/journal.pone.0223151)
Supplement: S1 Table — USA, United States of America; COPD, chronic obstructive pulmonary disease; ICU, intensive care unit; SICU, surgical intensive care unit; MICU, medical intensive care unit; A, < 24 h after mechanical ventilation; B, 24–48 h after mechanical ventilation; C, 48–72 h after mechanical ventilation; D, 72–96 h after mechanical ventilation; E, > 96 h after mechanical ventilation; F, > 5 days after ICU admission; G, > 7 days after ICU admission; H, usual care; MRC, Medical Research Council; 1, ICU acquired weakness; 2, duration of mechanical ventilation; 3, length of ICU stay. (DOCX) [file pone.0223151.s008.docx]

Table 1 Basic characteristics of included studies

| Study | Country | ICU types and characteristics | Age (years) | | Sample size (male/female) | | Intervention time | | Outcomes |
| --- | --- | --- | --- | --- | --- | --- | --- | --- | --- |
|  |  |  | Control | Intervention | Control | Intervention | Control | Intervention |  |
| Hodgson et al., 2016 [18] | Australia, New Zealand | ICU adults | 53 ± 15 | 64 ± 12 | 21 (9/12) | 29 (21/8) | H | B | 2, 3 |
| Schaller et al., 2016 [19] | Austria, Germany, USA | SICU adults | 64 (45–76) | 66 (48–73) | 96 (61/35) | 104 (65/39) | H | B | 1, 3 |
| Schweickert et al., 2009 [4] | USA | ICU adults | 54.4 (46.5–66.4) | 57.7 (36.3–69.1) | 55 (33/22) | 49 (20/29) | H | C | 1, 2, 3 |
| Dong et al., 2014 [20] | China | ICU adults | 55.5 ± 16.2 | 55.3 ± 16.1 | 30 (20/10) | 30 (21/19) | H | C | 2, 3 |
| Moss et al., 2016 [11] | USA | ICU ARDS adults | 49 ± 15 | 56 ± 14 | 61 (35/26) | 59 (36/23) | H | E | 2, 3 |
| Burtin et al., 2009 [21] | Belgium | SICU and MICU surgical adults | 57 ± 17 | 56 ± 16 | 36 (26/10) | 31 (22/9) | H | G | 2, 3 |
| Denehy et al., 2013 [22] | Australia | ICU adults | 60.1 ± 15.8 | 61.4 ± 15.9 | 76 (52/24) | 74 (43/31) | H | F | 1, 2, 3 |
| Morris et al., 2008 [10] | USA | ICU adults | 55.4 ± 16.8 | 54.0 ± 16.8 | 165 (88/77) | 165 (93/72) | H | B | 2, 3 |
| Zhang et al., 2017 [23] | China | ICU COPD adults | 61.6 ± 5.6 | | 40 | 40 | H | D | 1 |
| Yu et al., 2016 [24] | China | ICU adults | 65.6 ± 15.2 | | 22 | 23 | H | C | 2, 3 |
| Hu et al., 2014 [25] | China | ICU adults | 65 ± 6.1 | | 37 | 43 | H | C | 2 |
| Shao, 2015 [26] | China | ICU adults | 52.71 ± 17.24 | 53.96 ± 16.41 | 77 (47/30) | 79 (47/32) | H | B | 2, 3 |
| Ke and Huang, 2016 [27] | China | ICU COPD adults | 54.2 ± 14.0 | 54.4 ± 12.7 | 50 (27/23) | 50 (26/24) | H | C | 1, 2, 3 |
| Huang et al., 2016 [28] | China | ICU adults | 54.23 ± 14.0 | 54.45 ± 12.67 | 50 (27/23) | 50 (26/24) | H | A | 1, 2, 3 |
| Yu, 2013 [29] | China | ICU adults | 55.74 ± 17.0 | 58.48 ± 17.4 | 42 (22/20) | 42 (19/23) | H | C | 1, 2, 3 |

USA, United States of America; COPD, chronic obstructive pulmonary disease; ICU, intensive care unit; SICU, surgical intensive care unit; MICU, medical intensive care unit; A, < 24 h after mechanical ventilation; B, 24–48 h after mechanical ventilation; C, 48–72 h after mechanical ventilation; D, 72–96 h after mechanical ventilation; E, > 96 h after mechanical ventilation; F, > 5 days after ICU admission; G, > 7 days after ICU admission; H, usual care; MRC, Medical Research Council; 1, ICU acquired weakness; 2, duration of mechanical ventilation; 3, length of ICU stay.
