# Supplementary material for: What is the optimum time for initiation of early mobilization in mechanically ventilated patients? A network meta-analysis
Source: PLoS One. 2019 Oct 7;14(10):e0223151. doi: 10.1371/journal.pone.0223151 (PMC6779259; doi:10.1371/journal.pone.0223151)
Supplement: S2 Table — SUCRA, surface under the cumulative ranking; ICU,Intensive care unit;—, not mentioned. (DOCX) [file pone.0223151.s009.docx]

Table 2 SUCRA and Meanrank results

| Intervention time | SUCRA | | Meanrank | |
| --- | --- | --- | --- | --- |
|  | ICU-AW | Duration of mechanical ventilation | ICU-AW | Duration of mechanical ventilation |
| Time after mechanical ventilation |  |  |  |  |
| ≤ 24 h | 0.7 | 0.7 | 2.1 | 3 |
| 24–48 h | 0.2 | 0.6 | 4.1 | 3.3 |
| 48–72 h | 0.6 | 0.8 | 2.4 | 2 |
| 72–96 h | 0.8 | — | 1.8 | — |
| > 96 h | — | 0.4 | — | 4.4 |
| Time after ICU admission |  |  |  |  |
| > 5 days | — | 0.1 | — | 6.3 |
| > 7 days | — | 0.6 | — | 3.6 |
| Usual care | 0.1 | 0.3 | 4.5 | 5.4 |

SUCRA, surface under the cumulative ranking; ICU,Intensive care unit; —, not mentioned
